# Family Factors Associated With Excessive Online Activity Among Adolescents: A Latent Class Analysis of Cross-Sectional Data

**DOI:** 10.5964/ejop.16907

**Published:** 2026-05-29

**Authors:** Anastasios Fotiou, Eleftheria Kanavou, Myrto Stavrou, Maria Christopoulou, Panteleimon Voitsidis, Maria Lappa, Clive Richardson, Anna Kokkevi

**Affiliations:** 1 University Mental Health, Neurosciences, & Precision Medicine Research Institute, “COSTAS STEFANIS” (UMHRI), Athens, Greece; 2 Department of Psychology, Panteion University of Social & Political Sciences, Athens, Greece; 3 First Department of Psychiatry, Medical School, Aristotelian University of Thessaloniki, Thessaloniki, Greece; 4 Medical School, National & Kapodistrian University of Athens, Athens, Greece; 5 Panteion University of Social & Political Sciences, Athens, Greece; Birmingham City University, Birmingham, United Kingdom

**Keywords:** excessive Internet use, adolescents, family correlates, latent class analysis, Greece

## Abstract

Adolescents’ online engagement offers both opportunities and risks: whereas moderate use can foster learning, social connection, and entertainment, excessive use has been associated with sleep problems, lower academic performance, and psychosocial difficulties. The family context plays a central role in shaping adolescents’ daily routines, including online behaviours, through support, monitoring, and the quality of parent–child relationships. Despite growing concern about compulsive Internet use, evidence linking family factors to distinct patterns of online activity remains limited. This study examined the association between family characteristics and typologies of adolescent Internet use. Latent class analysis was conducted on data from a nationally representative sample of 3,202 16-year-old students in Greece to determine distinct classes of adolescents based on the frequencies of six online activities. Four classes of Internet users were identified: “heavy-with-gaming/gambling”, with high probability of very frequent involvement in all Internet-related activities (14.3%, mostly boys); “heavy-without-gaming/gambling” (44.1%, mostly girls); “moderate” (21.4%, mostly boys); and “light” users (20.3%, mostly girls). Compared with the group of “light” Internet users, the “heavy-with” and “heavy-without-gaming/gambling” groups had seven times and twice the odds, respectively, of reporting compulsive Internet use according to the Compulsive Internet Use Scale score. Multinomial multivariable logistic regression testing for associations with family variables showed that low satisfaction with the relationship with the father was associated with membership of the class of “heavy-with-gaming/gambling” users. Interventions tailored to the specific needs of adolescents engaging in heavy online activity, particularly when including gaming and gambling, should take into account the adolescent–father relationship.

The Internet has become an intrinsic part of adolescents’ daily lives in most parts of the world (ITU, 2023). Adolescents rely on the Internet to produce and consume recreational content, play games, communicate and interact with peers, search for popular and school-related material, shop and sell goods, and express themselves and engage socio-politically (Bitto Urbanova et al., 2023; Chassiakos & Stager, 2020; Khalaf et al., 2023). The Internet may also function as a compensatory virtual community where adolescents can fulfil their need to form new online and offline relationships or maintain existing ones, find mutual support and warmth, and escape from anxieties (Cheah et al., 2022; Kardefelt-Winther, 2014; Popat & Tarrant, 2023; Rideout & Fox, 2018).

While the Internet offers opportunities for personal and social engagement and development, very frequent engagement may interfere with the daily functioning and wellbeing of some adolescents, posing challenges to their somatic and mental health (Firth et al., 2019; Hoare et al., 2016; Prizant-Passal et al., 2016). At the same time, recent evidence highlights that effects are heterogeneous: digital engagement can produce both risks and benefits, with small average negative associations but also a range of positive outcomes such as enhanced social connection, creativity, and socio-emotional development (Haddock et al., 2022; Orben, 2020). Thus, whether intensive use supports or undermines wellbeing appears to depend strongly on the type and quality of activities, as well as individual and contextual factors. Against this mixed background, studies have pointed out that excessive use of social media may be associated with anxiety, increased feelings of depression, poor body image, reduced self-esteem, and loneliness (Bozzola et al., 2022; Kwak et al., 2022; McCrae et al., 2017; Yurdagül et al., 2021); persistent online gaming has been associated with serious interference in daily social and academic life, loss of creativity, increased sedentary behaviour and related harm, psychological distress, and negative psychological symptoms (Cheng et al., 2018; Derevensky et al., 2019; Parker, 2019); and online gambling has been linked with alcohol use, violence, delinquency, and high-risk sexual activity (Floros, 2018). Overall, it is not the engagement itself but the pattern of excessive or uncontrolled use that may be regarded as a behaviour that shares features with addictions, with potentially enduring adverse consequences for adolescent development (Firth et al., 2019; Pluhar et al., 2019).

As with other behaviours that either promote or present risks to health, the salience of the Internet in adolescents’ lives depends profoundly on family characteristics and processes. Adolescents typically begin and consolidate Internet use within their unique family environments. A large body of empirical research has uncovered the independent, direct or indirect role of family characteristics in excessive and particularly problematic Internet use among adolescents (see, for example, Ding et al., 2017; Faltýnková et al., 2020; Fischer-Grote et al., 2019; Petruzelka et al., 2020; Schneider et al., 2017). Evidence reliably implicates factors such as family structure (e.g., restructured families), low socioeconomic status, family functioning and climate, and especially parent–adolescent relationships (lack of common social activities, parental rejection, low parental support and care), parenting styles (authoritarian parenting, lack of rules), and parental digital practices (gaming, gambling).

So far, most studies that have examined the role of family characteristics in adolescent problem Internet use have relied on approaching their target population by treating adolescents as if they form a homogeneous group, typically defined by comparatively higher scores on relevant Internet addiction scales (hence, ‘problem’ Internet users). In doing so, existing studies tend to ignore the possibility that — in different contexts — the category of ‘problem Internet users’ may consist of more nuanced groups of adolescents with distinctive personal attributes, including their Internet use preferences (gaming, social media, gambling, pornography, etc.) and the characteristics of their proximal or distal environments.

Only a few studies have analysed adolescent Internet use by first identifying subgroups based on common preferences and frequency of use and then examining how these patterns relate to family characteristics. A German study of 14- to 17-year-olds (*n* = 1,744) applied latent profile analysis to the Compulsive Internet Use Scale (CIUS), a 14-item measure of problematic Internet use, and identified five groups, one of which (3.2% of the sample) showed a profile consistent with pathological use (Wartberg et al., 2015). Membership of this group was associated with poor family functioning, high family conflict, and lower life satisfaction. A Spanish study used a questionnaire on attitudes towards interactive media and frequency of Internet use to derive latent groups (Rial et al., 2015). It identified four groups, including one that used the Internet for more than two hours daily with no parental control, a profile which was also independently associated with a higher frequency of arguments with parents over Internet use. In the UK, Eynon and Malmberg (2011) relied on survey items covering five types of Internet activities — communication, information seeking, entertainment, participation, and creativity — to classify 8- to 19-year-olds (*n* = 1,069) into four distinct profiles. Having parents who set rules about Internet use emerged as a predictor of group membership across all profiles — including among those making proactive use of the Internet (such as creating and communicating online content) — and especially among high-frequency users. Taken together, these studies demonstrate the potential of latent class/profile analysis to capture heterogeneous patterns of adolescent Internet use and to link them with aspects of family context. Yet, their scope is relatively narrow, often limited to general indicators of family functioning, parental control, or conflict. Other important dimensions — such as parental support, satisfaction with parental relationships, socioeconomic conditions, parental employment, or parental monitoring — were not systematically considered, thus providing only a partial view of how family structures and resources intersect with different patterns of adolescent Internet use.

Similarly to these three studies, the present study asserts that a more nuanced inspection of adolescents who use the Internet is warranted when seeking meaningful associations with familial characteristics. Unlike these studies, however, the present work further seeks to classify its population into groups based on the frequency of Internet use combined with their online preferences. Specifically, our study draws on a large nationally representative sample of 16-year-old students in Greece and uses mixture modelling with the aim of identifying latent subgroups of adolescents who share particular attributes regarding their online preferences and frequency of engagement. It then explores associations between membership of the different subgroups and family characteristics, especially with regard to relations with the mother and father separately.

The present study employs a relatively loose term (‘heavy’) to describe higher-risk Internet use, a behaviour that may significantly interfere with several aspects of an adolescent’s life at home and school (Lissak, 2018). Here, “heavy use” is employed descriptively to indicate high frequency or intensity of online engagement. Following reviews that underline the coexistence of risks and opportunities (Haddock et al., 2022; Orben, 2020), this term does not imply that the underlying activities are necessarily negative but rather that frequent engagement — whether in beneficial or problematic pursuits — warrants closer scrutiny. Although it may be considered to convey a negative connotation, namely loss of control, the term is preferred to the comparatively stricter “excessive”, “compulsive”, “problem” or “pathological” use. We made no a priori hypotheses about the number of classes of adolescent Internet users, but we expected to identify at least one subgroup characterised by heavy use of multiple online activities, including online gaming. Drawing on the extensive literature on family correlates, we also expected to find that adverse family conditions — such as restructured family, low economic status, low parental control, and poor relationships with parents — would be independently associated with a higher probability of membership of the subgroup(s) of adolescents who make heavy use of the Internet.

## Method

### Sample

We used data from the Greek arm of the 2015 European School Survey Project on Alcohol and Other Drugs (ESPAD, www.espad.org), conducted by the Athens University Mental Health, Neurosciences, & Precision Medicine Research Institute (ESPAD Group, 2016). The target population was 16-year-old high school students (Grade 10). A nationwide stratified, clustered probability sample was drawn from the Ministry of Education’s list of schools, with the school class as the primary sampling unit. Stratification took into account geographical region and school type (private/public, comprehensive/vocational).

Of the 1,558 schools in the sampling frame, 175 were contacted and 167 (95.4%) participated. Self-completion, pencil-and-paper questionnaires were group-administered by trained research assistants to students in the sampled classes during school hours. Absences on the day of administration accounted for 7.0% of enrolled students, and 2.1% of those present either refused participation or lacked parental permission. Of the 3,507 questionnaires collected, 305 (8.7%) were excluded according to ESPAD data-cleaning rules, leaving 3,202 cases for analysis.

### Measures

#### Internet Use Measures

Respondents were asked on how many days in the past week they had used the Internet for each of six activities: communicating on social media; playing online games; gambling; reading or searching for information; streaming or downloading music, videos, or films; and selling, buying, or searching for products. Because the distributions of days of use were highly skewed — or, in the case of reading or searching for information, bimodal — it was decided to treat these measures as categorical. To avoid low frequencies (and, consequently, failure of the estimation procedure in the latent class models), responses were recoded into three groups, as far as possible of approximately equal size. If the relative frequency of the highest or lowest response category was greater than one-third, this extreme category formed one group, and the remaining categories were dichotomised into two approximately equal groups. The final categories were: social media, 0–2 days/3–6 days/7 days; gaming and gambling, 0/1–3/4–7; reading or searching for information, 0–1/2–3/4–7; streaming and downloading, 0–3/4–6/7; and selling, buying, or searching for products, 0/1/2–7.

Participants also completed the Compulsive Internet Use Scale (CIUS; Meerkerk et al., 2009, 2010), which measures loss of control, preoccupation, withdrawal symptoms, coping, and conflict concerning Internet use. Fourteen items (for example, “How often do you find it difficult to stop using the Internet when you are online?”) are answered on a five-point Likert scale (0 = “Never” to 4 = “Very often”), giving total scores from 0 to 56. The CIUS showed high internal consistency (Cronbach’s alpha = .902). Following Meerkerk et al. (2009, 2010), individual scores were dichotomised into compulsive (score > 28) and non-compulsive Internet use (≤ 28).

#### Family Correlates

Parental support was measured by asking how readily adolescents received warmth, caring, and emotional support from the mother and father, separately. Responses ranged from “Almost always” to “Almost never” on five-point Likert scales and were recoded as “High parental support” (both answers “Almost always”) and “Low parental support” (all other responses).

Students were asked how satisfied they usually were with their relationships with their mother and father, separately. The response options — five on a scale from “Very satisfied” to “Not at all satisfied” and, additionally, “There is no such person” — were dichotomised into “High satisfaction” if the response was “Satisfied” or “Very satisfied”, and “Low satisfaction” otherwise (including responses indicating “There is no such person”; 21 cases [0.7%] for mother and 122 [3.8%] for father in the total sample).

Satisfaction with the family’s financial situation was measured on a five-point Likert scale from “Very satisfied” to “Not at all satisfied”. Responses of “Satisfied” or “Very satisfied” were recoded as “High satisfaction”, and all other responses as “Low satisfaction”.

Students were asked who else lived in their household and were recorded as “living with both biological parents” (yes/no), depending on whether they nominated both father and mother or not. Students who lived with father or stepfather and mother or stepmother were classified as “Living with both parents”, and the rest as “Living with a single parent”.

Responses to the question on the country of birth of the student’s parents were classified as “Both parents born in Greece” or “At least one parent not born in Greece”.

Participants were asked about the employment status of their father and mother, separately. Possible responses were: “Yes, Employed”; “No: Retired, Income from other source, Disability, or Taking care of the household”; “No, Seeking work”; and “Don’t know/Don’t have/Don’t see”. Responses for both items were dichotomised into “Employed” (only the response “Yes”) and “Not employed” (all other responses).

Students also estimated how well off their family was compared with other families in the country, with seven response categories from “Very much better off” to “Very much less well off”. These were dichotomised into “Better off” (collapsing responses from “Better off” to “Very much better off”) and “Not better off” (responses from “About the same” to “Very much less well off”).

Parental monitoring was measured by asking students if their parents knew where they spent their Saturday nights. The four response options, from “Always know” to “Usually don’t know”, were dichotomised into “High parental monitoring” (“Always know”) and “Low parental monitoring” (all other responses).

The questions on parental employment status, ethnicity, and satisfaction with the family’s financial situation were national additions to the ESPAD questionnaire.

### Statistical Analyses

Statistical analysis was conducted using the Complex Samples module in IBM SPSS, Version 20.0 to take into account the stratified, clustered sampling design and case weights. The Pearson product–moment correlation coefficient was used to assess correlations between the numbers of days per week involving different Internet activities. Pearson’s chi-square test and Student’s *t*-test (via the GLM procedure) were used to compare categorical and scale variables, respectively, between genders. Listwise deletion of missing data was adopted for all analyses.

Latent class analysis (LCA) models were fitted to identify patterns of use across the six online activities investigated, using the *R* package poLCA (Linzer & Lewis, 2011). Models with one to five classes were fitted to determine the optimal number of classes, based on conceptual considerations and several statistical criteria, including Akaike’s Information Criterion (AIC), the Bayesian Information Criterion (BIC), sample size–adjusted BIC (a-BIC), G^2^ fit statistic, and entropy (Linzer & Lewis, 2011). Models were fitted both with and without gender as a covariate. In both cases, the optimal number of classes identified was four. Since both the literature and our preliminary analyses indicated that types of Internet use are gender-sensitive, the four-class model including gender as a covariate was retained for further analysis. Posterior probabilities from this model were used to classify students into latent classes, applying the maximum probability assignment rule, which assigns individuals to the class with the highest posterior probability of membership (Bray et al., 2015).

The association between latent classes and CIUS status (compulsive/non-compulsive Internet use) was tested using a logistic regression model, with CIUS status as the dependent variable and gender and latent classes as explanatory variables. The associations between latent classes and family variables were subsequently tested in a multinomial logit regression model, with latent classes as the dependent variable and again controlling for gender. Class membership in this second model was calculated from the posterior probabilities of an inclusive LCA with the optimal number of classes, including gender and all family variables as covariates. Inclusive LCA was conducted to eliminate bias (Bray et al., 2015). This approach was not adopted for the relation between CIUS status and latent classes (the first model) due to the high correlation between them. Similarly to logistic regression, multinomial logit regression fits models for the ratios of the probabilities of belonging to each of the categories of the dependent variable, compared with the probability of belonging to the reference category. However, these relative probabilities are not odds, as in logistic regression, because the two probabilities involved are not complementary. Consequently, we refer to the exponentiated regression coefficients — which indicate the multiplicative effects of each explanatory variable on these relative probabilities — as ‘relative probability ratios’ rather than odds ratios. The data supporting the findings of this study are freely available in the Open Science Framework (OSF) at Fotiou (2026).

## Results

### Internet Use and Family Characteristics

The sample for analysis consisted of 3,202 adolescent students (50.8% female), with a mean age of 15.7 years (range 15.3–16.2). Table 1 presents data on Internet use and the prevalence of adverse family characteristics, for the total sample and by gender. Nearly 70% of participants reported using the Internet every day in the week preceding the survey. Average use was 5.8 days (standard deviation [*SD*] = 2.1). The average number of days of Internet use ranged from less than one day per week for gambling and for selling/buying or searching for products, to more than five days per week for communicating on social media.

**Table 1:** Descriptive Statistics for Internet Use and Family Variables in the Total Sample and by Gender

	% or *M*(*SD*)			
	Total	Male	Female	
Variable	*N* = 3,202	*N* = 1,576	*N* = 1,626	*p*
Internet Use Variable				
Percentage using the Internet every day ^a^	68.7	65.4	72.0	.002
Average number of days using Internet ^a^	5.8 (2.1)	5.6 (2.2)	5.9 (2.0)	.009
Average Number of Days ^a^ Using the Internet for:				
• Communicating on social media	5.5 (2.4)	5.1 (2.5)	5.8 (2.2)	< .001
• Gaming	1.4 (2.2)	2.5 (2.6)	0.3 (1.0)	< .001
• Gambling	0.3 (1.0)	0.5 (1.4)	0.1 (0.5)	< .001
• Reading, surfing, searching for information	2.9 (2.3)	2.8 (2.3)	3.0 (2.2)	.041
• Streaming/downloading music, videos, films	4.5 (2.3)	4.3 (2.4)	4.7 (2.3)	< .001
• Selling/buying or searching for products	0.6 (1.3)	0.7 (1.5)	0.5 (1.1)	< .001
High CIUS ^b^ score (>28/56)	15.1	11.0	19.0	< .001
Family Variable				
Low parental support	36.0	40.4	31.8	< .001
Not satisfied with relationship with the father ^c^	18.8	14.5	23.0	< .001
Not satisfied with relationship with the mother ^c^	9.6	7.7	11.4	.004
Not satisfied with family's financial situation	29.7	26.1	33.1	< .001
Not living with both biological parents	15.4	15.4	15.4	.967
Living with a single parent ^d^	12.6	13.3	11.8	.270
At least one parent not born in Greece	19.8	19.4	20.3	.628
Father not employed ^e^	17.3	17.4	17.3	.887
Mother not employed ^e^	32.3	30.7	33.9	.093
Family not better-off compared to other families	52.9	53.0	52.8	.878
Low parental monitoring	30.6	36.5	24.8	< .001

*Note.* ^a^ In the past seven days. ^b^ Compulsive Internet Use Scale. ^c^ Cases of “No such person” are classified with “Not satisfied” (Mother: 21 cases; Father: 122). ^d^ Sixty cases reporting no parents were included as “Single parent”. ^e^ Cases of “Don't know”, “Don't have” or “No contact” are included in the analysis with “Not employed” (Mother: 34 cases; Father: 111).

The numbers of days per week across the various Internet activities were positively correlated with each other and statistically significant at *p* ≤ .001, except for the use of social media with gaming (*p* = .030) and with gambling (*p* = .47). Overall, significantly more females (72.0%) than males (65.4%) reported daily use of the Internet, whereas males reported higher rates of gaming, gambling, and selling/buying or searching for products. A significantly higher percentage of females (19.0%) also scored high on the CIUS, compared with males (11.0%).

### Latent Class Analysis of Internet Use

**Figure 1:**
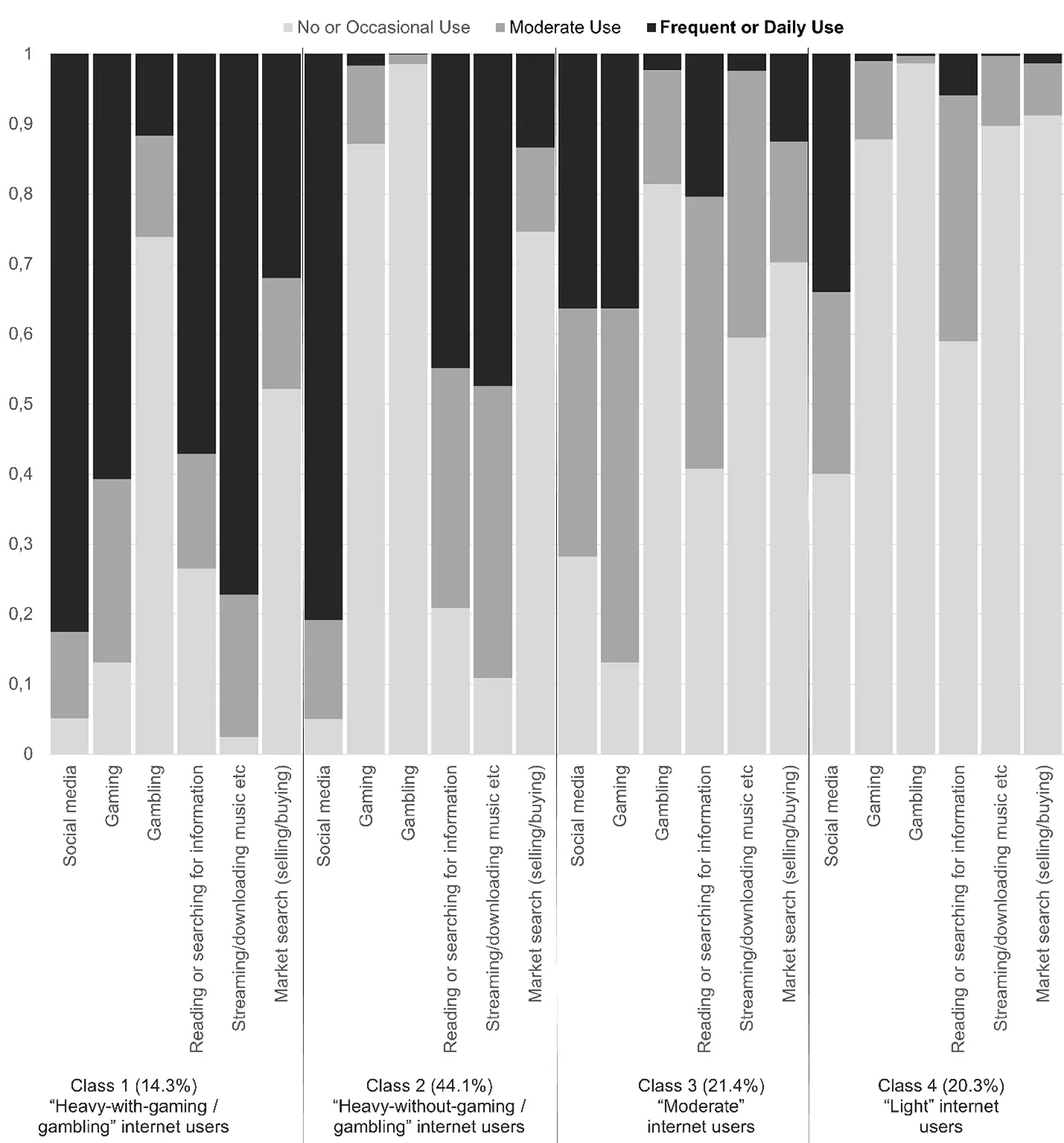
Conditional Item Response Probabilities for Each Latent Class *Note*. “No or occasional use” = 0–2 days for social media, 0 days for gaming, gambling, and market search, 0–1 days for reading or searching for information, and 0–3 days for streaming/downloading music, etc.; “Moderate use” = 3–6 days for social media, 1–3 days for gaming and gambling, 2–3 days for reading or searching for information, 4–6 days for streaming/downloading music, etc., and 1 day for market search; “Frequent or daily use” = 7 days for social media and streaming/downloading music, etc., 4–7 days for gaming, gambling, and reading or searching for information, and 2–7 days for market search.

The best-fitting LCA model including gender, as indicated by the lowest values of the BIC and corrected AIC, comprised four classes (see Table 2). Figure 1 presents the conditional item response probabilities within each latent class. Adolescents in the first class — labelled “heavy-with-gaming/gambling” users — had the highest probabilities of reporting frequent use of the Internet for every activity and made up 14.3% of the sample. The second class — “heavy-without-gaming/gambling” users (44.1% of the sample) — consisted of adolescents with relatively high probabilities of reporting all types of Internet use except gaming and gambling. Adolescents in the third class — “moderate” users (21.4%) — reported moderate to frequent use of several types of Internet activity, including gaming but neither gambling nor streaming/downloading. The fourth class — “light” Internet users (20.3%) — included adolescents with high probabilities of reporting moderate or frequent use of social media and moderate use of reading, surfing, and searching for information, but low probabilities of using the Internet for other activities.

**Table 2:** Latent Class Analysis

Number of classes	Log-likelihood	Residual *df*	BIC	Adjusted BIC	cAIC	G^2^	Entropy
1	-16300.69	716	32698.05	31341.59	32710.05	2441.12	-
2	-15607.38	702	31424.20	31341.59	31450.20	2121.41	.70
3	-15190.10	688	30702.44	30575.34	30742.44	1195.23	.71
4	-15022.24	674	30479.49	30307.91	30533.49	935.73	.71
5	-15023.66	660	30595.11	30379.05	30663.11	1003.15	.76

*Note.* Indices of fit for models with varying numbers of classes, including gender as a covariate (*df* = degrees of freedom; BIC = Bayesian Information Criterion; cAIC = corrected Akaike Information Criterion; G^2^ = likelihood ratio chi-square).

Boys had a higher probability than girls of belonging to the “heavy-with-gaming/gambling” class (.295 for boys versus .033 for girls) and the “moderate” Internet user class (.410 for boys versus < .001 for girls) (not shown in Table). Girls had a higher probability than boys of belonging to the “heavy-without-gaming/gambling” (.698 versus .158) and “light” (.269 versus .137) classes.

### Latent Class Membership and High CIUS Status

Logistic regression analyses for CIUS status by latent class (not shown in Table) showed that membership of the “heavy-with-gaming/gambling” Internet user class (Class 1) was associated with a sevenfold higher probability of compulsive Internet use [gender-adjusted odds ratio (aOR) and corresponding 99% confidence interval (CI): aOR = 6.6, 99% CI = 3.7–11.7], compared with “light” Internet users. Membership of the “heavy-without-gaming/gambling” (Class 2) and “moderate” Internet user classes (Class 3) was associated with probabilities of compulsive Internet use increased by approximately two- and threefold, respectively (aOR = 2.0, 99% CI = 1.3–3.3; and aOR = 2.7, 99% CI = 1.4–5.0), compared with “light” Internet users.

### Latent Class Membership and Family Characteristics

Table 3 presents the results of multinomial logistic regression analyses of latent class membership in relation to family characteristics. Values of goodness-of-fit statistics (pseudo *R*^2^) were .472 (Cox and Snell) and .510 (Nagelkerke). Adolescents in households with low parental monitoring were twice as likely to be “heavy” Internet users (with or without gambling/gaming) [Relative Probability Ratio (RPR)_Class1_ = 2.0, 99% CI = 1.3–3.2 and RPR_Class2_ = 1.8, 99% CI = 1.2–2.6)] and 60% more likely to be “moderate” Internet users (RPR_Class3_ = 1.6, 99% CI = 1.0–2.3) than “light” users. Students with low satisfaction in their relationship with their father were less likely to be “heavy-without-gaming/gambling” or “moderate” users (RPR_Class2_ = 0.5, 99% CI = 0.2–0.9 and RPR_Class3_ = 0.5, 99% CI = 0.3–0.9, respectively) than “heavy-with-gaming/gambling” users.

**Table 3:** Results (Relative Probability Ratios and 99% Confidence Intervals) of Multinomial Logistic Regression Analysis of Latent Class Membership in Relation to Family Correlates (*N* = 3,081)

		RPR [99% *CI*]					
		Reference class category: ‘Light' users ^a^			Reference class category: ‘Heavy’ users ^a^		
Family characteristics	*p*	‘Heavy-with gaming / gambling’	‘Heavy-no gaming / gambling’	‘Moderate’	‘Heavy-no gaming / gambling’	‘Moderate’	‘Light’
Low parental support	.155	1.2 [0.8, 1.9]	0.9 [0.6, 1.3]	1.3 [0.9, 2.1]	0.7 [0.5, 1.1]	1.1 [0.7, 1.6]	0.8 [0.5, 1.2]
Not satisfied with relationship with the father	.001	1.6 [0.8, 3.2]	0.7 [0.5, 1.1]	0.8 [0.4, 1.7]	**0.5 [0.2, 0.9]^b^**	**0.5 [0.3, 0.9]**	0.6 [0.3, 1.2]
Not satisfied with relationship with the mother	.332	1.4 [0.6, 3.4]	1.4 [0.8, 2.5]	1.2 [0.5, 2.8]	1.0 [0.5, 2.2]	0.9 [0.4, 1.7]	0.7 [0.3, 1.6]
Not satisfied with family’s financial situation	.021	1.0 [0.6, 1.7]	1.5 [1.0, 2.2]	1.1 [0.7, 1.8]	1.5 [0.9, 2.4]	1.1 [0.7, 1.7]	1.0 [0.6, 1.8]
Not living with both biological parents	.454	0.9 [0.2, 5.6]	1.5 [0.7, 3.5]	1.5 [0.3, 6.8]	1.6 [0.4, 6.8]	1.5 [0.4, 5.4]	1.1 [0.2, 6.2]
Living with a single parent	.767	1.4 [0.2, 10.6]	1.4 [0.6, 3.5]	1.5 [0.3, 8.6]	1.1 [0.2, 6.0]	1.1 [0.3, 4.4]	0.7 [0.1, 5.8]
Both parents [nationality]	.016	0.6 [0.3, 1.1]	1.1 [0.8, 1.5]	0.6 [0.4, 1.0]	1.8 [1.0, 3.1]	1.1 [0.7, 1.6]	1.7 [0.9, 2.9]
Employed father	.644	1.2 [0.7, 1.9]	1.0 [0.7, 1.6]	1.3 [0.8, 2.2]	0.9 [0.5, 1.5]	1.1 [0.7, 1.7]	0.9 [0.5, 1.4]
Employed mother	.219	1.0 [0.7, 1.5]	1.2 [0.9., 1.6]	0.9 [0.6, 1.3]	1.2 [0.8, 1.9]	0.9 [0.6, 1.3]	1.0 [0.7, 1.5]
Family better-off compared to other families	.044	1.0 [0.7, 1.5]	1.4 [1.0, 1.9]	1.1 [0.7, 1.6]	1.4 [0.9, 2.1]	1.1 [0.8, 1.4]	1.0 [0.7, 1.5]
Low parental monitoring	< .001	**2.0 [1.3, 3.2]**	**1.8 [1.2, 2.6]**	**1.6 [1.0, 2.3]**	0.9 [0.6, 1.4]	0.8 [0.5, 1.1]	**0.5 [0.3, 0.8]**

*Note.* RPR = Relative probability ratios; *CI* = Confidence intervals. ^a^ Gender was also included in the model as a control variable; **^b^** Boldface denotes statistically significant associations.

## Discussion

Drawing on data from a large, nationally representative sample of 16-year-old students in Greece, the present study sought to identify subpopulations of adolescents distinguished by their online behaviour and to examine the association between group membership and family characteristics. Four groups of adolescent Internet users were identified on the basis of the frequency of multiple online activities, including two characterised by excessive Internet use. One group, comprising mostly boys, was characterised by heavy use of the Internet across all activities, including online gaming and gambling. The second group, consisting mostly of girls, was defined by heavy use of four activities but with a much lower probability of online gaming and gambling. Compared with the group of “light” Internet users, these two groups had sevenfold and twofold odds, respectively, of reporting compulsive Internet use (according to the CIUS score). A poor relationship with the father was the only family characteristic distinguishing the “heavy-with-gaming/gambling” group from the other three groups of Internet users.

Our findings highlight two issues, each with potentially significant implications for prevention. First, adolescents who use the Internet excessively are not a homogeneous group in terms of either gender or preferred online activities. Although our study had no a priori hypotheses regarding the number of latent classes, we expected to identify at least one class of adolescents making excessive use of the Internet. Only a few previous studies have explored different groups of adolescent Internet users based on their digital preferences and frequency of engagement (Eynon & Malmberg, 2011; Rial et al., 2015; Wartberg et al., 2015), and only two of these identified ‘heavy’ (Rial et al., 2015) or ‘high-risk’ (Wartberg et al., 2015) groups. However, none of these studies identified higher-risk Internet user groups while also accounting for online gaming and/or gambling behaviour.

Our analyses identified two distinct groups of excessive Internet users, differentiated by their probabilities of engaging in online gaming and gambling. With the rise of the gaming and gambling industries, and the opportunities these offer for concurrent digital social interaction, adolescents — particularly in the age cohort studied here — have mastered the art of building communities in and around video and gambling competitions (Lenhart, 2015). Increasingly, adolescent gamers or gamblers not only compete with digital others but also form sincere and lasting companionships with peers they know yet have limited opportunities to meet in person (Kowert & Kaye, 2018). This presents novel and promising opportunities for healthy social engagement and interaction among adolescents. Positive aspects aside, however, the combined use of gaming, gambling, and social media may be cause for concern. In addition to the adverse effects of excessive social media and online communication, members of this group may also face the specific health and psychosocial risks associated with gaming and gambling. This finding makes a meaningful contribution to prevention and harm reduction, as it suggests that gaming and gambling may cluster within the daily routines of a substantial group of adolescents who spend much of their extracurricular time online.

Latent youth communities characterised by heavy Internet use also differed in terms of gender composition. As our analyses showed, adolescents with high probabilities of reporting every online activity, including gaming and gambling, were mainly boys. In contrast, girls had a higher probability of belonging to the “heavy-no-gaming/gambling” group. Internet activity in this group included heavy use of social media and online communication platforms which — although also popular among boys — are more frequently used by girls. Interestingly, membership of this class was associated with twice the probability of compulsive use status.

Although much research supports the finding that boys tend towards gaming whereas girls spend more of their time on social media (e.g., Dong et al., 2018; Kelly et al., 2019; Twenge & Martin, 2020), our finding is important in highlighting the need for a gendered approach to promoting health-informed Internet use in this population. Specifically for boys, excessive gaming has been found to share common characteristics with the use of psychotropic substances (see Weinstein & Lejoyeux, 2015, for a review), and gambling has been linked with a range of adverse health behaviours and conditions, including deviance and substance use (Floros, 2018; Petry et al., 2018), as well as more severe medical and psychiatric problems in adulthood (Potenza et al., 2019). For girls, the use of social media has been associated with depressive symptoms and lower overall wellbeing (Kelly et al., 2019; Twenge & Martin, 2020).

Secondly, heavy Internet use that includes online gaming and gambling may be associated with the quality of the relationship with the father. In our data, a poor father–child relationship was the only familial factor linked with an increased probability of membership of the “heavy-with-gaming/gambling” group, compared with the “heavy-without-gaming/gambling” and “moderate” Internet user groups. This finding is consistent with a German study that also used latent profile analysis and showed that the high-risk Internet user group displayed lower levels of family functioning and more problems in family interactions (Wartberg et al., 2015).

Elsewhere, several studies have established that a poor relationship with parents — and, more broadly, poor family functioning and support, negative parenting attitudes, and confrontational relationships over Internet use — are associated with adolescent problematic use and Internet addiction (e.g., Sela et al., 2020; Wartberg et al., 2014; see also Nielsen et al., 2019, for review). A poor relationship with parents may act as a major stressor, particularly for emotionally more vulnerable adolescents (Trumello et al., 2021; Wang et al., 2018), who may consequently turn to the Internet as a coping mechanism to relieve stress (Radovic et al., 2017) and to manage emotional or behavioural problems arising from strained parent–adolescent relationships.

Recent longitudinal evidence suggests that parents may respond ineffectively to adolescents who engage in heavy Internet use, which in turn may exacerbate the need for further engagement (Koning et al., 2018; Lin et al., 2020). A poor relationship specifically with the father has been associated with excessive Internet use in at least four previous studies (Lei & Wu, 2007; Li et al., 2022; Liu et al., 2013; Park et al., 2008). Furthermore, in two prospective studies, father–adolescent bonding predicted lower rates of problem online gaming at follow-up (Choo et al., 2015; Su et al., 2018; but see also Kim et al., 2018). Unlike at least one other study (Park et al., 2014; although similar to Su et al., 2018), the present analysis found that membership of the “heavy” Internet user group was not associated with the quality of the relationship with the mother. This may be explained by the different roles that mothers and fathers typically play in the digital lives of adolescents, with mothers more often engaged in monitoring and guidance, whereas the quality of the father–child relationship may exert its influence through broader emotional and relational mechanisms rather than direct supervision. Fathers are also reported to worry more than mothers about the dangers of Internet use and may therefore engage in increased monitoring and psychological control of adolescents’ Internet behaviour (Wang et al., 2005), which could in turn fuel adolescents’ need for further digital engagement (Lin et al., 2020).

### Limitations

Among the strengths of the present study is its use of mixture modelling to classify adolescents into latent groups of Internet users with differentiated profiles relating to the frequency of their online activity and the role of family factors. However, the study is not without limitations. First, the entropy level of .71 in the present study may arguably be considered to indicate some uncertainty in class separation. Entropy values closer to 1 indicate clearer demarcation of classes (Celeux & Soromenho, 1996). Although no generally agreed cut-offs exist, a rule of thumb is that values above .80 may be regarded as indicating acceptable entropy. In this respect, the entropy level reached in the present study suggests that at least some of the classes can be reasonably well differentiated.

A key limitation is the cross-sectional design of the research, which allows for the detection of associations but not the determination of ætiology or temporality between heavy Internet use and family functioning. Therefore, we were unable to establish whether a poor relationship with the father preceded or followed adolescents’ heavy use of the Internet. Importantly, our research design could not capture the complex and, in many respects, bidirectional effects of heavy Internet use among adolescents and the Internet-specific attitudes and behaviours of their parents (e.g., Koning et al., 2018; Lin et al., 2020).

In addition, as per standard procedure (Guttormsson et al., 2015), we used self-report questionnaires for data collection. This means that the data for both the dependent and explanatory variables were drawn from the same respondents, thereby increasing the risk of systematic measurement error that could account for a considerable proportion of the observed variance (known as common method variance; Podsakoff et al., 2012). However, it should be noted that the items measuring the key constructs in our study — for example, the relationship with mother or father — were not perceptually linked to Internet use, were presented in formats different from those measuring Internet use and appeared in a separate part of the questionnaire. All of these features are thought to help mitigate bias.

An additional limitation of the present study concerns the nature of the measures used. As with all analyses based on large-scale surveys such as ESPAD, constructs could not be assessed in depth due to constraints of space and scope. Measures of internet use frequency relied on simple self-reports of days of use per activity, and although the CIUS is psychometrically robust, the appropriate cut-off point for dichotomisation among adolescent internet users remains under debate (see, e.g., Laconi et al., 2014). The > 28 threshold was applied for reasons of comparability and interpretability. Similarly, family-related variables were measured through single items, which provide only a limited perspective on the broader family context. These measures should therefore be considered simplistic proxies. Nevertheless, retaining individual family items rather than aggregating them into a composite variable preserved detail and allowed identification of which specific family aspects were most relevant to compulsive internet use.

Another limitation is the lack of detail in the study regarding the devices that adolescents use to access the Internet, ranging from PCs to mobile devices such as smartphones, as these may affect not only the type of activity undertaken (Jeong et al., 2016) but also, very likely, the level of parental monitoring. Our findings should also be considered in the context of patterns of family management, parenting, and Internet use that are specific to Greece. Heavy Internet use is growing rapidly with the expansion of wireless access to fast and inexpensive Internet and with the proliferation of devices through which adolescents can go online. However, Greece remains among the European countries with the lowest levels of access to, and daily use of, the Internet (Eurostat, 2024). Replication of these findings with large, cross-national samples is important in order to enhance generalisability.

### Conclusion

The present study extends the limited body of research that has sought to identify latent subgroups of adolescents who share common Internet use attributes and to examine how membership of these groups relates to family characteristics such as family support, quality of parental relationships, and parental monitoring. We identified two groups of adolescents characterised by higher-risk Internet use. These groups may warrant interventions that are flexible and tailored to their particular characteristics: boys who engage in frequent gaming/gambling together with online communication, and girls who engage in frequent online communication. Our results also suggest that poor quality of the father–adolescent relationship is associated with heavy Internet use which includes online gaming and gambling. Family-focused prevention programmes and interventions may therefore be needed to enhance family functioning and strengthen parent–child relationships, with particular emphasis on the dynamic interactions between adolescents and their fathers. Fathers, too, need support to become more actively involved in their children’s online behaviour.

## Supplementary Materials

**Table:** 

Type of supplementary material	Availability/Access
Data	
01-Internet CIUS Paper - DATA FILE	Fotiou (2026)
Code	
02-Internet CIUS Paper - SYNTAX 4 ANALYSES	Fotiou (2026)
02-Internet CIUS Paper - SYNTAX 4 LCA	Fotiou (2026)
Material	
04-Internet CIUS Paper - ITEMS IN ENGLISH & NATIONAL LANGUAGE	Fotiou (2026)
Study/Analysis preregistration	
Study was not preregistered	—
Other	
03-Internet CIUS Paper - CODE BOOK-VARIABLES	Fotiou (2026)

## Data Availability

The data supporting the findings of this study are freely available in the Open Science Framework (OSF) at Fotiou (2026).
